# A qualitative study on the multi-level process of resilience development for adults recovering from eating disorders

**DOI:** 10.1186/s40337-021-00422-8

**Published:** 2021-06-09

**Authors:** Katie Grogan, Hannah O’Daly, Jessica Bramham, Mary Scriven, Caroline Maher, Amanda Fitzgerald

**Affiliations:** 1 School of Psychology, University College Dublin, Dublin, Ireland; 2 Elm Mount Unit, St. Vincent’s University Hospital, Dublin, Ireland

**Keywords:** Eating disorders, Anorexia nervosa, Bulimia nervosa, Binge-eating disorder, Resilience, Adults, Psychological well-being, Recovery

## Abstract

**Background:**

Resilience research to date has been criticised for its consideration of resilience as a personal trait instead of a process, and for identifying individual factors related to resilience with no consideration of the ecological context. The overall aim of the current study was to explore the multi-level process through which adults recovering from EDs develop resilience, from the perspectives of clients and clinicians. The objective of this research was to outline the stages involved in the process of developing resilience, which might help to inform families and services in how best to support adults with EDs during their recovery.

**Method:**

Thirty participants (15 clients; 15 clinicians) took part in semi-structured interviews, and responded to questions relating to factors associated with resilience. Using an inductive approach, data were analysed using reflexive thematic analysis.

**Results:**

The overarching theme which described the process of developing resilience was ‘Bouncing back to being me’, which involved three stages: ‘Who am I without my ED?’, ‘My eating disorder does not define me’, and ‘I no longer need my eating disorder’. Twenty sub-themes were identified as being involved in this resilience process, thirteen of which required multi-level involvement.

**Conclusion:**

This qualitative study provided a multi-level resilience framework for adults recovering from eating disorders, that is based on the experiences of adults with eating disorders and their treating clinicians. This framework provided empirical evidence that resilience is an ecological process involving an interaction between internal and external factors occurring between adults with eating disorder and their most immediate environments (i.e. family and social).

**Plain English summary:**

Anorexia nervosa, bulimia nervosa and binge-eating disorder demonstrate high rates of symptom persistence across time and poor prognosis for a significant proportion of individuals affected by these disorders, including health complications and increased risk of mortality. Many researchers have attempted to explore how to improve recovery outcomes for this population. Eating disorder experts have emphasised the need to focus not only on the weight indicators and eating behaviours that sustain the eating disorder during recovery, but also on the psychological well-being of the person recovering. One way to achieve this is to focus on resilience, which was identified as a fundamental aspect of eating disorder recovery in previous research. This study conceptualises resilience as a dynamic process that is influenced not only at a personal level but also through the environment in which the person lives. This study gathered data from adults with eating disorders and their treating clinicians, to devise a framework for resilience development for adults recovering from eating disorders. The paper discussed ways in which these findings and the framework identified can be easily implemented in clinical practice to facilitate a better understanding of eating disorder resilience and to enhance recovery outcomes.

**Supplementary Information:**

The online version contains supplementary material available at 10.1186/s40337-021-00422-8.

## Background

Anorexia nervosa (AN), bulimia nervosa (BN) and binge-eating disorder (BED) are three of the most commonly occurring ‘feeding and eating disorders’ for adults (DSM-V; American Psychiatric [1]). Eating disorder (ED) psychopathology in terms of AN, BN and BED involves biological (e.g. weight status), cognitive (e.g. over-evaluation of weight and appearance), affective (e.g. fear of eating in public) and behavioural (e.g. fasting, purging) features [2]. Despite the demonstrated efficacy of transdiagnostic ED interventions such as CBT-E [3, 4] or interpersonal psychotherapy [5, 6], as well as other ED subtype specific interventions such as Maudsley model of AN treatment for adolescents and young adults [7], epidemiological data demonstrates that recovery rates remain at approximately 55–69% [8, 9]. Even when eating behaviours improve, people with EDs can experience both short-term health issues regarding refeeding syndrome as well as long-term negative health outcomes such as cardiovascular conditions and osteoporosis [10–12].

Many researchers have attempted to explore how to improve recovery outcomes for this population. There are two main recovery paradigms that define recovery differently; the medical model and the recovery model. In comparison to the medical model, recovery is considered within the recovery model as a journey and a process, whereby the goals of recovery are determined by the individual rather than professionals [13, 14]. Due to the major health risks involved in EDs, professionals working with people with EDs may be at risk of over-emphasising the significance of biological and behavioural symptoms of EDs as the sole indicators of recovery, with a lack of consideration for cognitive and affective symptoms which may persist despite alleviation of the more observable symptoms [15].

ED recovery has been recognised as being difficult to achieve due to the ego-syntonic nature of the disorder [16], meaning that some people with EDs value their disorder and endorse their eating behaviours. A systematic review and qualitative meta-analysis were conducted by de Vos et al. [17], which aimed to collate findings from studies assessing fundamental criteria for ED recovery. The authors demonstrated that resilience was one of six criteria found to have large frequency effect sizes, which accounted for 13.8% of all recovery criteria assessed [17]. The authors concluded that resilience along with other elements of psychological well-being should be considered fundamental aspects of ED recovery. Other authors suggested that a focus on the psychological aspects of recovery would reduce the risk of ‘partial recovery’ occurring, whereby the physical and behavioural symptoms reduce but the psychological aspects remain [18], a term referred to by Keski-Rahkonen & Tozzi [19] as ‘pseudorecovery’ (pp. S83). The role of resilience in recovery is that it assists a person to better adapt to the stress and adversity they are experiencing and to gain control over their difficulties [14]. In this sense, it can be understood why individual factors such as self-efficacy and competence have been identified as “assets” influencing a person’s resilience; however, researchers have also identified that “resources” or external factors such as family support or community services also impact a person’s resilience (pp. 6, [20]).

Resilience, a concept defined within positive psychology perspectives [21] and commonly referred to as the ability to ‘bounce back’ from difficult experiences [22], might help to explain why some people with EDs recover well whilst others do not. According to Windle [20], who references the work of resilience theorists such as Luthar, Rutter, Ungar and Masten, resilience can be underpinned by the following features: (i) Resilience is influenced by factors beyond that of the individual, (ii) resilience is a process, and (iii) resilience occurs in response to adversity, stress or trauma. In relation to the first feature, human development is shaped by many environmental and societal influences [23]. In this sense, it may not be useful to identify personal, family and social factors in isolation, but rather to investigate the factors influencing resilience across personal, family and social levels (i.e. multi-level factors). Many experts in the resilience field acknowledge the importance of looking beyond individual traits [20, 24–26] to explain ‘psychosocial resilience’ [27] including within ED research [28]. In relation to the second feature, experts have highlighted the importance of assessing responses to stress and adversity as close as possible to the occurrence of the stressor so as to shift the focus of resilience research to stress reactivity rather than restoration of well-being [29]. This suggests that researchers should be trying to understand the process of resilience during ED recovery as it occurs, rather than when the adult has ‘recovered’. In relation to the final feature, the stress, trauma or adversity preceding resilience for people with EDs might include psychosocial difficulties related to the ED diagnosis [30], subsequent health complications [12], as well as other life adversities (e.g. negative familial comments about eating, weight or appearance [31, 32]).

Within the ED literature, two studies were identified which directly assessed resilience levels of adults with EDs [33]: the first demonstrated that resilience levels were lower for those recovering from EDs compared to those who had recovered from an ED and the general population [34]; and the second showed that increased resilience among those with EDs was associated with a reduction of ED symptoms, as well as improvements in psychological and social domains of quality of life over time [35]. Furthermore, Las Hayas et al. [36] used qualitative methods to identify the stages involved in the process of developing resilience for adults recovered from EDs, which included 14 stages. Of note, this was the first attempt within the ED literature to investigate resilience as a process, and the authors also identified that resilience facilitated the journey to recovery for people with EDs [36]. However, the authors noted a number of limitations to their study to be considered by future researchers.

This study set out to build on the current understanding of resilience as a process rather than a trait of a person, and to overcome some of the shortcomings of previous resilience research within the ED field by incorporating findings on the external influences associated with resilience alongside the personal influences. The overall aim of the current study was to explore the multi-level process through which adults recovering from EDs develop resilience, from the perspectives of clients and clinicians. The research question was ‘What are the personal, family and social influences on the process of developing resilience for adults recovering from EDs?’. The objective of this research was to outline the stages involved in the process of resilience development, which might help to inform families and services in how best to support adults with EDs during their recovery.

## Method

### Design

This qualitative study was conducted using a constructivist paradigm and a phenomenological approach, which aimed to address the research question by ascertaining the human experience of the resilience process during ED recovery from client and clinician perspectives. This approach acknowledged that resilience is a socially constructed concept and that the best means in which to explore this concept was via semi-structured interviews, with the view that in-depth qualitative research can help to guide good clinical practice as well as inform policy decisions [37]. Ethical approval for this study was granted by a public healthcare ethics committee in July 2018 (Ref: 130618KG) and by a city-based hospital in April 2019 (Ref: RS19–002).

### Participants

#### Clients

Fifteen participants were recruited to form this sample. Eligibility criteria to be met included that participants were aged 18 years or above; they must have received a diagnosis of AN, BN, BED, Other Specified Feeding or Eating Disorder (OSFED) or Unspecified Feeding or Eating Disorders (UFED) determined through team-based assessment using DSM-V criteria; they must have been attending adult mental health services as either inpatients or outpatients; and they must have been deemed by their treating clinician to be at a stage of recovery which suggests they are making active steps towards their recovery. Prochaska and DiClemente [38] and Prochaska et al. [39] define five stages of recovery within their transtheoretical stages of change model: pre-contemplation, contemplation, preparation, action and maintenance. Regarding the inclusion criterion relating to stage of recovery, participants identified by their treating clinician as being in the final two stages (i.e. action, maintenance) were deemed eligible to participate in this study.

#### Clinicians

This sample comprised of 15 clinicians who self-identified as working with adults with EDs. Ten worked in a general adult mental health outpatient service, whereas five worked in a specialist ED unit. There was no overlap among clinicians across these two settings. These clinicians had on average 12.10 years (*SD* = 8.42) of experience working in their respective services.

### Procedures

Convenience sampling was used as the heads of two ED services (i.e. general adult mental health service and specialist ED unit) were approached in order to ascertain interest in participating in the research, both of whom agreed to their services taking part. A short research pitch was prepared and delivered by the lead author (KG) whereby service staff members were allowed the opportunity to ask questions about the research before agreeing to participate. Information sheets were provided 2 weeks prior to participant informed consent being retrieved. Interview slots were arranged with clinicians who agreed to take part themselves, and clinicians also disseminated the information sheets to potential clients who met eligibility criteria. All participants were screened using eligibility criteria, and interviews were scheduled and took place in the client’s and clinician’s place of treatment and place of work, respectively.

Sample size was determined based on guidelines from the qualitative methodology literature (e.g. [40–42]). The two groups were included to generate more perspectives on the topic which would help to inform clinical practice, similar to previous resilience research [36, 43].

On the day of the interviews, participants from both groups completed short demographic information questionnaires (see Table 1) before taking part in a semi-structured interview conducted by the lead researcher (KG). Prior to the commencement of the interview, the purpose of the research was explained to participants (i.e. to explore the multi-level process through which adults recovering from EDs develop resilience) and limits of confidentiality were outlined. Participants were asked to describe what their understanding of the term resilience was, and this was further clarified with participants by outlining three key features through which resilience is currently conceptualised as per Windle [20].

**Table 1:** Demographic information on client and clinician groups

	Client group (*n* = 15)	Clinician group (n = 15)
Gender		
*Male*	2 (13%)	5 (33%)
*Female*	13 (87%)	10 (67%)
Ethnicity		
Caucasian	15 (100%)	15 (100%)
Age (in years)	28.67 (11.13)	42.60 (8.23)
Age range (in years)	19–54	32–58
Age Dx was received (in years)	21.00 (7.20)	
Years living with Dx	9.17 (9.51)	
Dx received		
*AN*	7 (47%)	
*BN*	5 (33%)	
*AN + BN*^a^	1 (7%)	
*UFED*	2 (13%)	
Living situation		
*Family of origin*	8 (52%)	
*Friends*	3 (20%)	
*Family of choice*	1 (7%)	
*Living alone*	1 (7%)	
*Single parent living with children*	1 (7%)	
*Family of origin and family of choice in same house*	1 (7%)	
Employment status		
*Student*	6 (40%)	
*Employed*	6 (40%)	
*Unemployed but otherwise occupied*^b^	3 (20%)	
Comorbid diagnoses^c^		
*Depressive disorder*	7	
*Anxiety disorder*	5	
*Borderline personality disorder*	3	
*Obsessive compulsive disorder*	2	
*Psychosis*	1	
Mental health discipline		
*Nursing*		5 (33%)
*Psychology*		4 (27%)
*Dietetics*		3 (20%)
*Psychiatry*		2 (13%)
*Social work*		1 (7%)

Data in years are in the form of mean (SD). All other data are in the form of n (%)

*Dx* Diagnosis, *AN* Anorexia nervosa, *BN* Bulimia nervosa, *BED* Binge-eating disorder, *UFED* Unspecified feeding or eating disorder

^a^AN + BN refers to participants who have received lifetime diagnoses of both disorders, referred to as “diagnostic cross-over” in the DSM-V (pp.347)

^b^Unemployed but otherwise occupied included individuals who care for family members, retirees and homemakers

^c^Percentages were not ascertained for individual comorbid disorders as some participants fell into more than one comorbid category. Eight participants from the client sample (53.3%) reported having one or more comorbid disorders

### Data collection

Data were collected by means of semi-structured interviews using an interview guide. Questions from this interview guide were informed by the resilience literature and reviewed by two clinicians who treat people with EDs. Clinicians were asked to respond based on their experiences of working with adults with EDs thus far in their careers. The average duration of interview was 46.48 min (*SD* = 11.82). Interviews were conducted between August 2018 to November 2019.

### Data analysis

Data were analysed using reflexive thematic analysis (TA) as outlined by Braun and Clarke [41, 44, 45]. Reflexive TA is a version of thematic analysis, which aims to identify patterns of meaning within the data, but with the recognition of the active role of the researchers in the knowledge production process (i.e. reflexivity). The researchers engaged in active reflection throughout the research process, which was aided by the use of a reflective research journal in order to enhance research credibility [46, 47].

The 30 interviews, which were conducted and audio-recorded by KG, were transcribed verbatim by HOD. Transcripts were then reviewed by KG. The lead author engaged in a process of familiarisation with and immersion in the data by listening to the audio-recordings while reading transcripts. Initial ideas and thoughts about potential codes and/or themes were noted before generating codes using NVivo [48], a qualitative data analysis computer software package. Although the research question was considered from a theoretical perspective (i.e. Bronfenbrenner’s ecological theory), data were analysed in an inductive manner whereby themes were constructed based on a data-driven approach using latent coding, representing the researcher’s explicit interpretation of implicit meaning of data. Multiple codes could be given to any specific selection of text. Initial themes were generated, which were later reviewed and refined. Twenty percent of the data were also coded by a second author (HOD). This step was conducted so that authors could discuss ideas and decide on how best to tell the story of the data, rather than for reliability checking, which is not endorsed within this research paradigm [41]. Braun and Clarke’s [45] definition of a theme was used (i.e. stories about particular patterns of shared meaning across the dataset). The entire process was non-linear, requiring movement back and forth between each step. Criteria outlined in two documents, Standards for Reporting Qualitative Research (SRQR [49];) and Braun and Clarke’s [44] 15-point Checklist of Good Criteria for Thematic Analysis, were met to ensure good quality reporting of results.

The component of the research question which aimed to examine the process through which adults recovering from EDs develop resilience was addressed by identifying the temporal sequencing of the stages involved in the resilience process. This was achieved by sequencing themes in the order in which they were described to have occurred for participants in general and in an order that made logical sense. The research question also aimed to identify the multi-level factors influencing the resilience process, which was addressed by distinguishing personal, family and social influences during the coding process. ‘Personal’, ‘family’ and ‘social’ were assigned as prefixes to all codes in order to specify influences of resilience on a personal, family or social level.

### Methodological rigour

Various strategies were utilised as per Lincoln and Guba’s [50] four trustworthiness criteria to ensure methodological rigour in the current study (see Additional Information 1 in the Supplementary Materials section). Considering the interpretivist approach within which this study took place, attempts at enhancing the trustworthiness of the study serve to develop more rich, in-depth and reflexive findings, rather than to achieve validity or reliability of results.

## Results

The following section describes the multi-level process through which adults recovering from EDs develop resilience. Samples of supporting quotations from participants can be found in Table 2. ‘Participants’ is the term used to refer to the client and clinician groups collectively, whereas findings specific to one or the other group are indicated by using the terms ‘clients’ or ‘clinicians’.

**Table 2:** Quotations supporting sub-theme components across personal, family and social levels

Theme/ sub-theme	Level	Quotation
Stage 1: ‘Who am I without my ED’		
*Theme 1: ED dependency*		
1a. ED is a source of coping	Personal	“I think with certain people’s resilience- for example, with my resilience, my coping mechanism or my activity for resilience was to binge and purge. And so that was my resilience against other things”, *Client 6, female, aged 21–30.*
1b. ED takes over	Personal	“I kind of see it as, it was taking over, you know, more than I would have liked it to have done”, *Client 7, female, aged 18–20*
1c. Secrecy, denial and avoidance	Personal	“I went through a period then of like purging. And a bit of bulimia probably. All secret and I didn’t really see it as a problem myself then”, *Client 2, female, aged 18–20.*
	Family	“And then sometimes the parents just are a little bit in denial or guilty and all kinds of feelings about it”, *Clinician 10, female, aged 41–50.*
*Theme 2: Other ways to cope*		
2a. Other skills	Personal	“For me it was recognising just setting small goals. Focusing on this day. And the plan for the week. You can get an idea of where you want to be but don’t focus on it. Don’t focus on that huge road. Just focus on this step”, *Client 15, female, aged 21–30.*
2b. Learning from the past	Personal	“But the week I came back, for that really bad week, I’m actually really grateful for it. ‘Cause I actually learned so much from it”, *Client 15, female, aged 21–30.*
*Theme 3: The question of letting go*		
3a. Fear of making change	Personal	“And I think maybe my anorexia was subconsciously saying to me *Well look, you’re never going to be able to succeed anyway. So let’s just totally fail”, Client 4, female, aged 51–60.*
3b. Introspection	Personal	“So it’s being more understanding of it and not OKing it but not beating yourself up over it either. ‘Cause it’s a mental thing”, *Client 15, female, aged 21–30.*
	Social	“I also think that counselling really helps understand. Just with the internal understanding of why my coping mechanism was necessary and why I picked it up and why I don’t need it anymore.”, *Client 6, female, aged 21–30.*
3c. Motivation and readiness for change	Personal	“Wanting to be there for themselves is a big factor. Because unless they want it for themselves- you can’t want recovery for somebody else. It doesn’t work that way”, *Clinician 11, female, aged 31–40.*
Stage 2: ‘My ED does not define me’		
*Theme 4: Seeing the bigger picture regarding EDs*		
4a. Knowledge and understanding about EDs	Personal/ Social	“Like that feeling misunderstood thing is huge. Now that I understand myself more, but feeling like that “I don’t understand myself, no one else understands me”. Whereas I feel like I’m working with [professionals] who really do”, *Client 15, female, aged 21–30.*
	Family	“So I guess going against the resilience is a lack of understanding as to what’s happening. The thoughts within the family, that *why is the person doing this to us and doing it to themselves?.* As opposed to seeing it like a separate kind of entity. You know, that this is an illness and it’s separate from the individual. The individual is still who they always were. Just the illness has taken over”, *Clinician 4, female, aged 31–40.*
4b. EDs involve more than eating behaviours and weight	Personal	“It’s not just changing your ED, it’s changing everything that comes with it. Because an ED is your mindset, you know what I mean”, *Client 15, female, aged 21–30.*
	Family	“So families I think sometimes can be... a little short sighted, in the sense of making it all about the problem behaviour as opposed to what else is going on beyond it”, *Clinician 8, male, aged 51–60.*
	Social	“I was trying to access HSE funding for inpatient treatment. And the psychiatrist he rang and he said to me on the phone, he goes *Oh your BMI is a lot higher than I thought it would be.* And I was like *Are you actually saying this? You should know better”, Client 12, female, aged 21–30.*
4c. The difficult road ahead	Personal	“I thought it was a quick fix. I was only gonna be here for a couple of months to a year and then I’d be out the door and no one was ever gonna see me again. I realised that this was more of a lifetime thing than a quick fix”, *Client 8, female, aged 21–30.*
	Family	“Families I think look for the quick fix. The person themselves, generally by the time they come to us, would have been struggling with it for quite some time”, *Clinician 3, female, aged 31–40.*
	Social	“People need to know in dealing with an ED that it’s not a straight line road. And recovery takes a long time. And I know that I’ll never be ‘recovered’. ‘Cause you’re never gonna recover from something like this. ‘Cause there’s always gonna be a trigger at some point in your life”, *Client 13, female, aged 21–30.*
4d. Managing emotions	Personal	“If they have easier ways to handle their distress, if they’ve learned personal ways to manage distress but also learned how to verbalise their distress, identify their emotions, that kind of thing, I think that would be of huge benefit in terms of developing personal resilience. Now, it’s not going to stop the development of EDs, but I do think it may improve the bounce back ability”, *Clinician 8, male, aged 51–60.*
	Family	“And I find now if I come to [mother] and I’m struggling, she won’t get involved but she’ll step back and be like *Ok, well what do you know that works for you that’ll help this.* I find our relationship is actually so much better now. So I think she is quite helpful”, *Client 1, female, aged 21–30.*
	Social	“‘Cause quite often an ED presents, it engenders a lot of anxiety in the patient, the family and the clinicians. So what seems to help resilience from a clinical point of view is having an understanding of the disorder, not feeling anxious about meeting people and having a set of tools that you can use to help the engagement with the client and to settle things down as quickly as possible”, *Clinician 5, male, aged 51–60.*
*Theme 5: Safety and security*		
5a. Secure base and positive relationships	Personal	“But like having someone there, [Name], who just wants the best for me and I know loves me, is a massive thing”, *Client 15, female, aged 21–30.*
	Family	“At the start I remember when they were trying to make me eat, like I’d have temper tantrums and scream things at them. But they didn’t scream back, they just sat there and tried to say the right thing. Like they didn’t leave me or give up on me. That helped as well I think, just they didn’t go away. Which was what my ED told me they would do”, *Client 2, female, aged 18–20.*
	Social	“[Partner] never judges. He’s never mentioned whether you’re overweight, underweight”, *Client 11, female, aged 41–50.*
5b. Communication and honesty	Personal	“And I’d be honest with her about my ED. Another thing I learned is, even with my dad and my brother, I try not lie. You’re hiding that behaviour, it’s a secret, try and be more honest. You have to stop lifting this little friend. You more have to push it out in to the world, take it out of the closet, you know”, *Client 15, female, aged 21–30.*
	Family	“Even with adults, when the family is maybe not their family home - as in they’ve moved out and they’re living by themselves independently- there still is this need for connect within the families. And I would see that the most resilient people are the people who can go to their families and connect with them at some level”, *Clinician 4, female, aged 31–40.*
	Social	“But every time there’s a problem I just come to [staff] like *This is what’s going through my head. What do I do?”, Client 5, female, aged 18–20.*
5c. Balancing autonomy and support	Personal	“But I needed to cut the cord. I needed to become more independent. ‘Cause I think with the ED especially- well I think with the mental health- you become too dependent on others”, *Client 15, female, aged 21–30.*
	Family	“So it’s very difficult for them because the ED acts in a child-like way at times. By not taking responsibility for eating, by not taking responsibility for various other things in their life- financial, whatever. So the mother and father do step in to that parental role even though they’re an adult. So you’re trying to get them to step back, give the person more autonomy. Yet at the same time be there to support them when they’re having their difficulties. So it’s how do they sit through that, not ignore it but at the same time not patronise them and take control. There’s a real balance there they really have to find”, *Clinician 13, female, aged 31–40.*
	Social	“So it’s having that ability to know when we should help and when to stand back is quite important as well”, *Clinician 5, male, aged 51–60.*
*Theme 6: Watching out for potential knocks along the way*		
6a. General life stress	Personal	“And then I failed an exam and then I failed it again and then it just- I think the best way I could describe it was it was like my life just went upside down. Yeah, I wasn’t sure what to do with it. And then at that point the weight just kind of went *pssshh* (crashing sound). Like it just fell off me”, *Client 12, female, aged 21–30.*
	Family	“My dad had lost his job the year before. So he was at home and my mum, who always worked part time - she’s a nurse - started working full time. So I started taking on a lot of the cooking and all that kind of stuff”, *Client 2, female, aged 18–20.*
	Social	“I think the other blocks are maybe stuff that’s going on outside of the therapy room that is overwhelming the person, that we have no control over. Personal circumstances you know. They can’t get out of the home they’re in, they can’t get out the - current life situation”, *Clinician 6, male, aged 41–50.*
6b. Judgmental environment	Personal	“And then I’m very anxious about studying and always have been quite perfectionistic. I was playing a lot of sport. Hockey and camogie. So I was training at least once a day”, *Client 2, female, aged 18–20.*
	Family	“It was a very academic orientated household and a very stress orientated household, very ‘worky’. So it was less relaxing and stuff like that. Yeah, it’s an interesting dynamic, it’s very tense going back now”, *Client 6, female, aged 21–30.*
	Social	“Sometimes I’ll find girls that come in, they’ll have a number of friends who also have EDs within their group and there is a competitiveness when you reach a certain stage”, *Clinician 9, male, aged 41–50.*
6c. Food and body image emphasis	Personal	“I kind of blame myself for every situation and I blame my weight for every problem”, *Client 10, female, aged 21–30.*
	Family	“And then I find an awful lot of daughters who start exercising with their dads. This is a big thing. Going running with their dads and then like, a kind of competitive type thing going on around exercise”, *Clinician 9, male, aged 41–50.*
	Social	“Social media I think. And a big one that comes up, maybe more with the chronic patients is say [TV programme about weight loss]. Like all the obesity talk. For them, they hear that and that’s all they hear. And they have the fear of you know, if they did anything different they’re just going to gain and gain and gain weight. So I suppose when they’re hearing all these messages, that it can be really difficult”, *Clinician 14, female, aged 31–40.*
Stage 3: I no longer need my ED		
*Theme 7: ‘The resilient me’*		
7a. Routine and normality	Personal	“The whole resilience thing. ‘Cause I think it is huge. With mental health, when you’re down, you can feel so alien to other people as well, that normality is not something that can be for you.” *Client 2, female, aged 18–20.*
	Social	“So you can say *Well you’ve college and we know you have a break at this time and we know you’ve lectures. So let’s organise your diet plan around that*”, *Clinician 13, female, aged 31–40.*
7b. Positive mindset and future outlook	Personal	“So paradoxically, this idea of mindset can work for you, if you’re working for recovery, but it can work against you, if you’re focused enough. *No I can work harder and I can survive. I’ll be the one that beats the odds. I’ll be the one that manages to be eternally, infinitely thin”*, *Clinician 6, male, aged 41–50.*

### The process through which adults recovering from EDs develop resilience

**Fig. 1:**
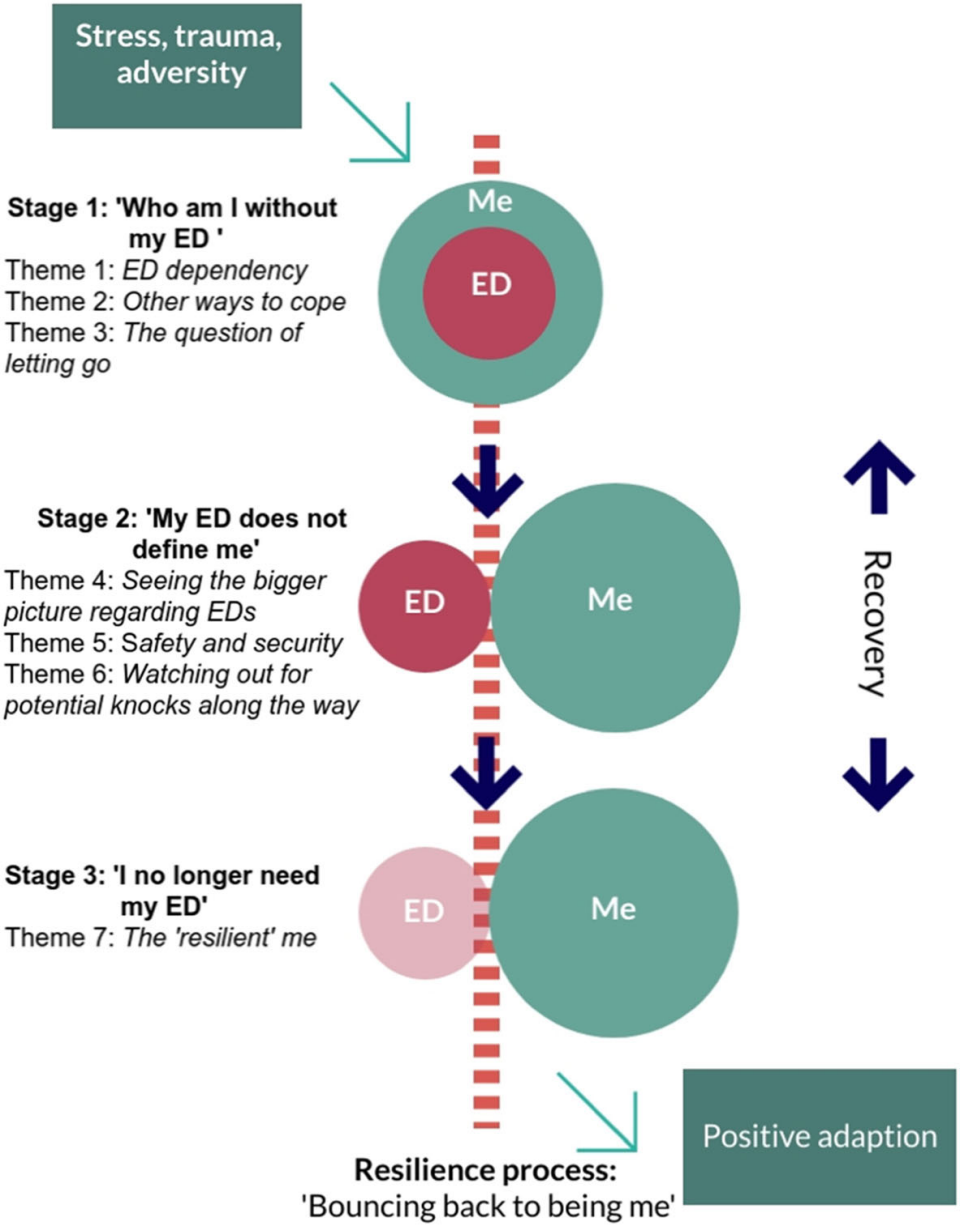
The process through which adults with EDs develop resilience during recovery

**Fig. 2:**
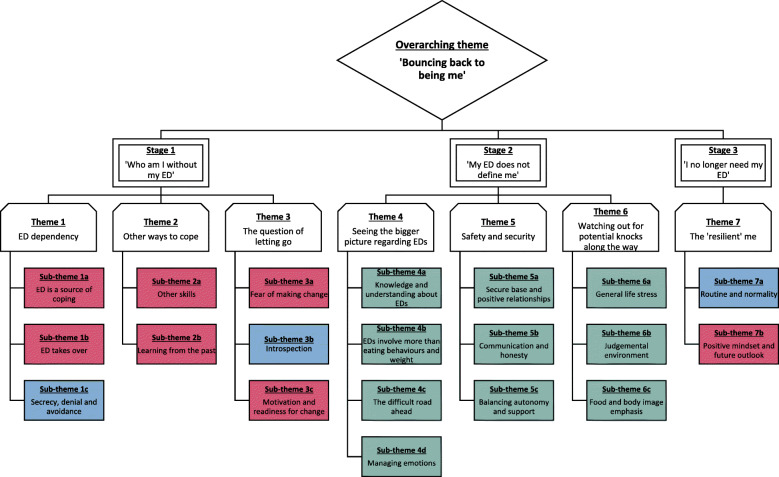
Thematic map of overarching theme, themes and sub-themes depicting the stages involved in the multi-level process of resilience development for adults recovering from EDs. Colour code: Red = sub-themes influencing resilience on a personal level; Blue = sub-themes influencing resilience on a personal and family/social level; Green = sub-themes influencing resilience on a personal, family and social level

The multi-level process of resilience development for adults recovering from EDs can be described under the overarching theme, ‘Bouncing back to being me’, which is best understood under three separate stages; Stage 1 (‘Who am I without my ED?’) includes three themes, stage 2 (‘My ED does not define me’) also includes three themes, and stage 3 (‘I no longer need my ED’) includes one theme (see Fig. 1). As can be seen in Fig. 2, there are 20 sub-themes involved in this process; seven sub-themes are influenced on an individual level, three are influenced on an individual and family/social level, whereas ten sub-themes are influenced on a personal, family and social level. This demonstrates the significance of the interaction between internal and external factors in the process through which adults recovering from EDs develop resilience. Below is a brief description of the resilience process for adults recovering from EDs. It is highly recommended that readers view Additional Information 2 from the Supplementary Materials section for the expanded interpretation of the results.

### Stage 1 of the resilience process: ‘Who am I without my ED?’

This stage describes a period whereby adults with EDs become defined by their ED, and are unable to separate their own identity to that of the ED (Stage 1: ‘Who am I without my ED?’). This stage generally reflects a personal process, with little involvement at a family or social level, except for sub-themes 1c and 3b.

During this initial stage, participants report that adults with EDs can feel quite dependent on their EDs in order to cope with the demands of the world and possibly to survive (Theme 1). Within this ED dependency theme, participants discuss how ED behaviours assist the adults with EDs to cope with stress or adversity (Sub-theme 1a); clients regularly discuss a point in their lives whereby the ED completely takes over (Sub-theme 1b) and becomes “all-consuming” *(Client 4, female, aged 51–60);* and how adults with EDs hide or deny their eating problems, or act in an avoidant manner regarding the severity of their eating issues (Sub-theme 1c). Family can also play a role in minimising or denying the ED issues which inevitably leads to the problem persisting rather than the person confronting the issue in a more proactive manner. Taken together, these three sub-themes reflect a stage whereby the adult with an ED becomes quite dependent on their ED in order to cope with the demands of the world and possibly to survive.

Adults with EDs often go through a period of considering other possible ways they might cope instead of depending on their ED (Theme 2). During this period, adults with EDs may pay more attention to their other skills (e.g. communication, problem-solving) which might assist them in coping with their difficulties (Sub-theme 2a); and clients also discuss the advantage of having experienced good coping previously in their lives, resulting in a learning from past experiences (Sub-theme 2b). These two sub-themes involving an acknowledgement of personal skills and evidence of having previously coped with life difficulties allow the adult with an ED to consider what other possible ways they might cope instead of depending on their ED as a core coping strategy.

Participants describe a period of time whereby adults with EDs weigh up the pros and cons of letting go of the ED (Theme 3). As noted previously, EDs assist some people to cope with life adversity, and so they may fear that letting go of their ED will have a negative impact on them (Sub-theme 3a); over time, adults with EDs develop more self-awareness through introspection (as well as through input from their family and service) about why the ED formed for them in the first instance (Sub-theme 3b); and they identify their motivations to let go of the ED and experience a readiness to let go (Sub-theme 3c). These three sub-themes involving initial fears of making change, introspection regarding why the ED formed in the first instance, as well as reflecting on personal motivation for change are all part of a process whereby the adult with an ED is weighing up the pros and cons of letting go of the ED.

### Stage 2 of the resilience process: ‘My ED does not define me’

During the second stage, the adult with ED begins building a self-identity separate to their ED by tapping into resources on an individual, family and social level (Stage 2: ‘My ED does not define me’). All sub-themes included in this stage are influenced across all three levels, reflecting the multi-level input in developing resilience. Though the adult with an ED may be less reliant on and less consumed by their ED compared to the first stage of the resilience process, the ED is still very much part of their life.

The resilience process is greatly promoted during this second stage when individuals with EDs, their families and other people in their immediate environment see the bigger picture regarding EDs (Theme 4). This theme highlights the importance of the individual, their family and service gaining knowledge and understanding about EDs so the client feels “understood” and “validated” (*Clinician 13, female, aged 31–40)* (Sub-theme 4a); the recognition by the individual, their family and service that the ED is not just about eating behaviours (i.e. dieting, bingeing etc.) and weight (Sub-theme 4b); the understanding of the individual, their family and service that the recovery journey is long and non-linear (Sub-theme 4c); and the importance of the person, their family and society in being able to manage their anxiety about the disorder (Sub-theme 4d). Collectively, this increased knowledge about EDs, the emphasis of factors beyond weight and eating behaviours in recovery, the knowledge that recovery is a long journey and the ability to manage anxieties about the disorder results in an ability to view the bigger picture of what is involved in ED diagnoses.

Safety and security (Theme 5) are noted as important aspects of the resilience process, which are influenced on an individual, family and social level. Participants emphasise the importance of having a secure base and positive relationships in building resilience (Sub-theme 5a); the need for openness and honesty between the adult with an ED and their family members (both about the ED and about other family issues) as well as the wider public (i.e. friends and service providers) (Sub-theme 5b); and the importance of striking a balance between providing support but also allowing the person to be autonomous and independent, especially regarding ED decisions (Sub-theme 5c). These sub-themes collectively result in a safe and secure environment in which the adult with ED can develop resilience.

Participants discuss the importance of being ready for and aware of potential knocks to their resilience through personal, family and social influences throughout the recovery process (Theme 6). As significant adversity or cumulative life stresses are often related to the onset of EDs for adults with EDs, other potential life stresses during recovery might trigger a worsening of symptoms or relapse (Sub-theme 6a); another potential knock to resilience to be aware of for people with EDs is judgemental environments in terms of pressure, high expectations and comparisons (Sub-theme 6b); and adults with EDs also appear susceptible to any factors that could potentially lead to a disturbed body image, which occurs when they are exposed to an over-emphasis on the importance of food and body image in society (Sub-theme 6c). These sub-themes of general life stress, judgemental environments and an emphasis on food and body image are areas which need to be monitored in order to prevent a set-back in the resilience process.

### Stage 3 of the resilience process: ‘I no longer need my ED’

During the final stage of the resilience process, adults with EDs no longer feel they need their ED in order to cope with the demands of the world or to survive due to better developed resilience and the utilisation of a wider set of resources (Stage 3: ‘I no longer need my ED’).

It is recognised that adults with EDs begin to identify resilience in themselves (Theme 7) and become aware that they no longer need the ED to cope and survive. Within ‘the resilient me’ stage, adults with ED are able to reintegrate “more normal experiences” *(Client 6, female, aged 21–30)* into their lives (Sub-theme 7a); and they may have developed a more positive mindset and future outlook on their life (Sub-theme 7b). This sub-theme includes personal factors that may be more traditionally perceived as ‘traits’ of resilience. Such factors include patience, determination, hope, high self-esteem, self-direction, and self-belief, all of which were noted by the participants as important factors leading to a more generally positive mindset. However, these factors are referred to as goals to work towards or characteristics to be built upon, and not factors that either exist or do not exist within a person. Once this positive mindset is achieved, a person is at a stage within the resilience process which better facilitates recovery (i.e. a reduction of ED symptoms, improved psychological well-being). However, clinicians warn that these personal factors can also work to maintain a person’s ED (i.e. self-determination to remain thin), and although adults with EDs might go on to recover successfully, some may otherwise be at risk of relapse. This highlights the dynamic process involved in developing resilience during ED recovery.

## Discussion

### Summary of research aims and findings

This qualitative study aimed to describe the multi-level process through which adults recovering from EDs develop resilience, from the perspectives of clients and clinicians working in the field. The overarching theme which described the process of developing resilience was ‘Bouncing back to being me’, which involved three stages: ‘Who am I without my ED?’, followed by ‘My ED does not define me’, and finally ‘I no longer need my ED’. This study demonstrated that self-identity was recognised as an important feature in this resilience process, whereby the resilience process involved a regaining of personal identity separate to that of the ED identity. Within ED research, Bowlby et al. [51] showed that recovery involves an understanding that the ED is separate from one’s identity as a person. This study built on these findings by identifying the various stages involved in this multi-level resilience process through which a person recovering from an ED develops a sense of self that is separate to that of the ED. This process might explain how EDs become less ego-syntonic, as the thoughts and behaviours associated with EDs are no longer as valuable to the person’s sense of self. As long as a person’s sense of self is intertwined with the ED, it is difficult for thoughts and behaviours related to the ED to become ego-dystonic. The separation between sense of self with that of the ED might make it easier to challenge disordered thoughts and behaviours which may be driving the EDs during treatment.

### Findings in relation to previous research

This study built on the previous research conducted by Las Hayas et al. [36], demonstrating many similarities across study findings. Of the 14 themes identified in their study, all themes overlapped conceptually with findings from the present study. However, the current study identified a number of themes which were not identified by Las Hayas et al. [36]. Firstly, this study referred to sub-themes occurring early on in the resilience process such as ‘ED is a source of coping’ and ‘Secrecy, denial and avoidance’. It is likely that this difference reflects the inclusion of participants in recovery in the current study which differed to Las Hayas et al.’s [36] inclusion of ‘recovered’ individuals only. As suggested previously, it is important to investigate responses to stress and adversity as close as possible to the occurrence of the stressor so as to shift the focus of resilience research to stress reactivity rather than restoration of well-being [29]. Therefore, it was valuable to include these sub-themes which reflect important influences relevant to the early stages of the resilience process, as specified by participants. Another unique sub-theme identified in the current study was the importance of ‘Learning from the past’. Participants discussed how the ability to reference a previous time in their life that they showed good coping, particularly during past difficult experiences, facilitated the resilience process. Exposure to adversity in moderation can initiate previously untapped resources, allowing a person to benefit from support they were not previously utilising, and this leads to mastery for future adversities experienced [52]. Finally, there were various sub-themes identified in stage two which were not demonstrated by Las Hayas et al. [36] including the acknowledgement of ‘The difficult road ahead’, the importance of ‘Managing emotions’, the significance of ‘Communication and honesty’ and the acknowledgement of the potential impacts of ‘Judgemental environments’ as well as ‘Food and body image emphasis’ on the resilience process. It is important to note that these sub-themes were identified as multi-level influences, meaning that they were significant across personal, family and social levels of influence. Factors beyond the individual were not addressed by Las Hayas et al. [36]. These findings support the postulation of experts in the field that resilience is a multi-level, psychosocial and ecological concept [24–27, 53], which was similarly emphasized by ED experts [28, 36].

### Clinical implications

Findings from the current study are relevant to clinicians who are working with adults recovering from EDs specifically. First, this division of the resilience process into three stages allows for the identification of the resilience stage a person is at during recovery. The identification of the three stages assists clinicians to pace the expectations of the client, their families and themselves. For instance, the process suggests that it would be unrealistic for clients to want to fully let go of their eating behaviours before the identification of other skills and coping abilities has occurred. This knowledge will help clinicians and clients to set realistic goals during treatment. The caveat of this resilience process, just like other psychological processes outlined in previous research, for example, the transtheoretical stages of change [38] or the five stages of grief [54], is that it is not as simplistic as it may appear [55]. This process, though outlined as a linear process for ease of access, is more dynamic and complex, and it is likely that some adults may skip steps, or steps may occur at different stages for different individuals. Without this consideration, clinicians may be at risk of over-simplifying a complex process [20, 56].

Second, this framework identified the multi-level influences on the development of resilience during ED recovery, which were most significant during the second stage of the resilience process. This multi-level process reduces the level of expectation and the burden put upon people in recovery to be solely responsible for their ‘bounce-backability’. Researchers in other areas of resilience research (e.g. disability) previously reported the potential danger in implying individual responsibility without consideration of external factors [57]. Results demonstrated that families, friends and services have a direct role in influencing the resilience process by understanding the bigger picture of what is involved with EDs, by creating a safe and secure environment within which the person can recover, and through a reduction in behaviours and attitudes that may result in potential knocks to the person’s resilience. Family-based interventions are often recommended in working with clients with EDs, especially considering family factors may inadvertently influence the recovery of adults with EDs (e.g. enabling behaviours, expressed emotions, psychological distress [58]).

Finally, the findings pertaining to this resilience process fit with positive psychology perspectives that aim to restore hope in individuals overcoming adversity by identifying what makes life worth living rather than to focus solely on alleviating symptoms [29, 59]. This supports personal recovery perspectives which aim to allow a person to function their best despite ongoing persistence of symptoms [60]. This multi-level resilience process similarly suggests that by building up one’s resilience (rather than focusing on symptom reduction), adults with EDs arrive at a point whereby they no longer depend on their ED to function or survive, suggesting that positive psychology interventions such as life coaching and resilience training may be useful in working therapeutically with adults with EDs. There has been some evidence of the efficacy of positive psychology interventions in the forms of ‘positivity groups’ [61] and mindfulness-based intervention [62] for adults with EDs, but research on such interventions appears to be far less frequent compared to the more commonly researched interventions such as CBT-E [3]. It must be recognised that though various psychological or psychotherapeutic interventions might serve as effective treatments for EDs, clinicians might be ethically obliged to focus on the ED behaviours if they pose a significant risk to the person (e.g. risk of starvation, cardiac problems).

### Research implications

From a research perspective, this study is one of four studies identified on the topic of resilience in the ED literature [34–36], and is the only study, to our knowledge, that focused on resilience from a multi-level perspective. This study provides support for a multi-level perspective of resilience, and sheds light on how future resilience research can look at an integrative approach by identifying factors influencing resilience across personal, family and social levels to establish the role of multi-level influences on resilience development.

### Strengths and limitations

As well as the strengths previously mentioned, this qualitative study offered unique contributions by allowing for the perspectives of adults with EDs as well as their treating clinicians to be captured through semi-structured interview, which has been suggested to be an effective means for guiding good clinical practice [37], particularly for a concept as complex as resilience [63]. As findings are based on client and clinician experiences, this increases the usability of the framework, which is more likely to be endorsed by clients and clinicians as it was informed by them. However, there were some limitations. Although we attempted to include male participants to avoid exclusion of this under-represented group, the client sample was not balanced for gender with only two males included. Furthermore, the framework might be more fully informed with the inclusion of data from family members, considering the role they appear to play in ED recovery [58].

## Conclusions

Despite these limitations, this qualitative study provides a rich description of the multi-level process of resilience development for adults recovering from EDs. This framework provides empirical evidence that resilience is an ecological process involving an interaction between internal and external influences, which involves a dynamic interplay between adults with ED and their most immediate environments (i.e. family and social). The inductive and qualitative nature in which this process was identified should result in a high level of acceptability of the framework in clinical practice by clients and clinicians, so that this framework can be used to inform treatment choices and recovery plans for adults recovering from EDs from a positive psychology perspective.

## Supplementary Information

**Additional file 1.**

## Abbreviations

AN: Anorexia nervosa
BED: Binge-eating disorder
BN: Bulimia nervosa
CBT-E: Enhanced - Cognitive Behaviour Therapy
DSM-V: Diagnostic and Statistical Manual of Mental Disorders, fifth edition
ED: Eating disorder
OSFED: Other Specified Feeding or Eating Disorder
SD: Standard deviation
SRQR: Standards for Reporting Qualitative Research
TA: thematic analysis
UFED: Unspecified feeding or eating disorder
