# Supplementary material for: A qualitative study on the multi-level process of resilience development for adults recovering from eating disorders
Source: J Eat Disord. 2021 Jun 9;9:66. doi: 10.1186/s40337-021-00422-8 (PMC8191215; doi:10.1186/s40337-021-00422-8)
Supplement: Supplementary file 1 — Additional file 1. [file 40337_2021_422_MOESM1_ESM.docx]

**Supplementary Materials**

**Additional Information 1:** Trustworthiness criteria (Lincoln and Guba, 1986) and strategies applied to ensure methodological rigour

| Trustworthiness criteria | Suggested strategies | Strategies applied |
| --- | --- | --- |
| Credibility | Prolonged engagement with data | Researchers spent quality time immersing themselves in the data during data collection, transcribing, coding and analysis stages. |
|  | Data collection triangulation | Data were collected from two samples, which resulted in a richer description of the research topic. |
|  | Researcher triangulation | Multiple researchers were involved in all stages of the research process, including participant recruitment, data collection, coding, analysis and reporting. |
|  | Peer debriefing | This was completed via consultation with authors of the paper, as well as other qualitative research colleagues who were not involved in the study. |
| Dependability | Rich description of study methods | Researchers ensured a rich description of study methods by following reporting checklist guidelines provided by Braun and Clarke (2006) and the SRQR (O’Brien et al., 2014). |
|  | Developing an audit trail | All decisions were tracked and documented through multiple documents throughout the research process, including the ethics application forms, study protocol, reflexive journal and peer debriefing meeting minutes. |
| Confirmability | Researcher reflexivity | Researchers engaged in reflexive practice throughout the research process. The main author explicitly wrote about the philosophical underpinning of the study, the use of reflexive practice and the position of the researcher in the research to become more aware of the potential influence of the researchers on the findings. |
| Transferability | Rich descriptions of the sample | Descriptive statistics were provided on the participants, so that readers could easily ascertain the membership of the sample. This would allow readers to assess the transferability of findings from this study to their site of practice. |

#### Additional Information 2: Expanded interpretation of results

#### Stage 1 of the resilience process: ‘Who am I without my ED?’

This stage describes a period whereby adults with EDs become defined by their ED, and are unable to separate their own identity to that of the ED (Stage 1: ‘Who am I without my ED?’). This stage generally reflects a personal process, with little involvement at a family or social level, except for sub-themes 1c and 3b.

During this initial stage, participants report that adults with EDs can feel quite dependent on their EDs in order to cope with the demands of the world and possibly to survive (Theme 1). Participants discuss how the ED often occurred in the context of stress and/or adversity, and that the ED behaviours assist the adults with EDs to cope with these difficulties (Sub-theme 1a), sometimes by providing a sense of control or else by means of eating to help regulate emotions (i.e. emotional eating). For some participants, the ED behaviours appear to be the only option for overcoming stress or adversity experienced in life to date and provide a sense of being able to cope for the person. Although the eating behaviours serve an obvious function in a person’s attempt to cope and survive initially following adversity, clients regularly discuss a point in their lives whereby the ED completely takes over (Sub-theme 1b) and becomes “all-consuming” *(Client 4, female, aged 51-60).* During this time, the clients describe having limited resources in terms of time or energy to carry on with other aspects of their everyday life. The ED often becomes part of who the person is, so much so that they find it difficult to separate their identity from that of the ED, and they struggle to imagine life without the ED. This ED dependency stage can become risky for adults with EDs if they hide or deny their eating problems, or act in an avoidant manner regarding the severity of their eating issues (Sub-theme 1c). Family can also play a role in minimising or denying the ED issues which inevitably leads to the problem persisting rather than the person confronting the issue in a more proactive manner. Taken together, these three sub-themes reflect a stage whereby the adult with an ED becomes quite dependent on their ED in order to cope with the demands of the world and possibly to survive.

Adults with EDs often go through a period of considering other possible ways they might cope instead of depending on their ED (Theme 2). During this period, adults with EDs may pay more attention to their other skills which might assist them in coping with their difficulties (Sub-theme 2a). For instance, they consider their social skills, communication skills, distraction, realistic goal setting and problem-solving ability. Reflecting on these other skills positively influences self-efficacy as the person considers alternative means for coping other than using the ED as a coping mechanism. Participants discuss that the ability to cope is generally easier when the adults with EDs can reference a previous time in their life that they showed good coping, and how this positively impacted resilience at the time. In this sense, they are learning from past experiences (Sub-theme 2b). Furthermore, when adults with EDs have gone through a period of having “hit rock bottom”, *(Client 9, female, aged 31-40),* this provides an experience that they may wish to avoid at all costs in the future. Some adults with EDs note that they were grateful for past negative experiences as they could avail of the opportunity to learn how to “bounce back”, (*Client 13, female, aged 21-30).* These two sub-themes involving an acknowledgement of personal skills and evidence of having previously coped with life difficulties allow the adult with an ED to consider what other possible ways they might cope instead of depending on their ED as a core coping strategy.

Participants describe a period of time whereby adults with EDs weigh up the pros and cons of letting go of the ED (Theme 3). As noted previously, EDs assist some people to cope with life adversity, and so they may fear that letting go of their ED will have a negative impact on them (Sub-theme 3a). Participants recognise that the change involved in letting go of the ED or of entering a new way of coping with life’s stresses might negatively impact on the person’s resilience initially. For this reason, clients themselves describe the internal conflict between wanting to live a new way without the ED but also feeling scared and vulnerable of making change, and fearing failure associated with loss of ED status or not being able to let go of the ED. Over time, adults with EDs develop more self-awareness through introspection about why the ED formed for them in the first instance (Sub-theme 3b). For instance, they learn that the eating behaviours are not good for them but that they serve a certain function. However, although it is important to understand the function of the ED behaviour, participants also discuss how it is important to not condone these behaviours. Participants also recognise the support of services and professionals in assisting with this self-awareness process, through psychoeducation and psychotherapy. Adults with EDs sometimes identify their motivations to let go of the ED and a readiness to let go (Sub-theme 3c). The reasons for individuals wanting change are diverse, ranging from health risks to wanting to change for other people, and are often referred to as “turning points*”, (Client 13, female, aged 21-30).* However, participants mention that an important aspect is that the instigation for change needed to come from within the individual and couldn’t be driven by the wishes of family members, for instance. These three sub-themes involving initial fears of making change, introspection regarding why the ED formed in the first instance, as well as reflecting on personal motivation for change are all part of a process whereby the adult with an ED is weighing up the pros and cons of letting go of the ED.

#### 3.3 Stage 2 of the resilience process: ‘My ED does not define me’

During the second stage, the adult with ED begins building a self-identity separate to their ED by tapping into resources on an individual, family and social level (Stage 2: ‘My ED does not define me’). All sub-themes included in this stage are influenced across all three levels, reflecting the multi-level input in developing resilience. Though the adult with an ED may be less reliant on and less consumed by their ED compared to the first stage of the resilience process, the ED is still very much part of their life.

The resilience process is greatly promoted during this second stage when individuals with EDs, their families and other people in their immediate environment see the bigger picture regarding EDs (Theme 4). Participants discuss the importance of gaining knowledge and understanding about EDs (Sub-theme 4a). Participants spoke at length about the importance of gaining knowledge about food and nutrition, knowing the ED facts versus the myths, and developing a shared understanding with family and services about the nature of the person’s individual symptoms. This helps a person to feel “understood” and “validated” (*Clinician 13, female, aged 31-40),* and also aids in the early detection of relapse, with the support of family and services. Another important factor that the individual, their family and service need to be aware of is that the ED is not just about eating behaviours (i.e. dieting, bingeing etc.) and weight (Sub-theme 4b), as most people are led to believe initially. Instead, it must be considered that an ED is a mental health problem, involving negative internal dialogues that some participants referred to as “voices” *(Client 6, female, aged 21-30, Client 13, female, aged 21-30),* as well as intense emotional experiences. A main point discussed is that family and services need to recognise that weight restoration does not equate to recovery so that continued support for cognitive and emotional symptoms can occur even after the person recovering achieves better weight and behavioural outcomes. Another aspect of EDs that the majority of participants refer to was that the recovery journey is long and non-linear, with participants highlighting that being prepared for this difficult or ‘bumpy’ journey (Sub-theme 4c) avoids disappointment if recovery goals are not attained immediately. Participants recognise the importance of family members, friends and services sharing this understanding, so that expectations can be managed appropriately by all involved in supporting the adult recovering. Participants also reference the need to consciously decide to recover every day of this journey, as the ED habits can otherwise re-emerge automatically. Generally, understanding the bigger picture of what is involved in EDs assists the person, their family and society in being able to manage their anxiety about the disorder (Sub-theme 4d), and subsequently this can boost the resilience within the person recovering. Clinicians distinctly discuss that within a service, anxiety around working with certain disorders due to lack of knowledge about the disorder can result in over-control within clinical practice (e.g. being over-precautious) which leads to worse outcomes for the person recovering. Similarly, for family members, the importance of “managing their anxiety about EDs” *(Clinician 5, male, aged 51-60)* in order to positively impact on the management of emotions for the person recovering was emphasised. Participants discuss that an inability of family members or other supportive figures to contain emotions, also referred to as “high expressed emotion” *(Clinician 15, female, aged 31-40),* is unhelpful. High expressed emotion involves support figures portraying emotional overinvolvement, sometimes in the form of criticism or hostility, in a way which they think may be helpful but is not. Collectively, this increased knowledge about EDs, the emphasis of factors beyond weight and eating behaviours in recovery, the knowledge that recovery is a long journey and the ability to manage anxieties about the disorder results in an ability to view the bigger picture of what is involved in ED diagnoses.

Safety and security (Theme 5) are noted as important aspects of the resilience process, which are influenced on an individual, family and social level. Participants emphasise the importance of having a secure base and positive relationships in building resilience (Sub-theme 5a). Secure attachment, stability within the household and a lack of chaos within the family help to maintain a secure base. Participants discuss how the support within a home should not feel dependent on how ‘good’ the person recovering is. Non-judgemental partners and friends help to foster this safe and secure environment also and these relationships seem to incentivise a person to keep pushing through difficult times. Participants emphasise the need for openness and honesty between the adult with an ED and their family members (both about the ED and about other family issues) as well as the wider public (i.e. friends and service providers) (Sub-theme 5b). This requires good communication skills, listening and showing interest as well as a level of trust and a sense of connection between all involved. In terms of family relationships, unconditional positive regard, relating to a secure base mentioned previously, ensures that the individual can communicate honestly about their recovery progress. However, although there is a strong emphasis within the data on supportive relationships and creating a safe environment for adults with EDs, participants also emphasise the importance of striking a balance between providing support but also allowing the person to be autonomous and independent, especially regarding ED decisions (Sub-theme 5c). Participants discuss how this is very difficult to achieve because oftentimes, the adults with EDs might still be living in the home of origin, along with parents and possibly siblings. Particularly if the adult with an ED is medically unwell, the family can become directive in telling the person what they should and should not be doing. Barriers to personal autonomy include parental over-protection and over-involvement, which are reported to occur for people with EDs despite being adults, as well as service over-reliance whereby adults with EDs become too reliant on and lacking in boundaries with service providers. On the other hand, autonomy is better achieved by giving ownership to the client and by empowering the person in their recovery journey. Together, these sub-themes which are influenced across multiple levels emphasise the importance of a secure base and positive relationships, the need for communication and honesty and the importance of balancing autonomy and support provided to the person in recovery. These sub-themes collectively result in a safe and secure environment in which the adult with ED can develop resilience.

Participants discuss the importance of being ready for and aware of potential knocks to their resilience through personal, family and social influences throughout the recovery process (Theme 6). As significant adversity or cumulative life stresses are often related to the onset of EDs for adults with EDs, other potential life stresses during recovery might trigger a worsening of symptoms or relapse (Sub-theme 6a). These life stresses could relate to issues with family, partners or friends, or even issues within the therapeutic relationship. Some participants acknowledge that when they are stressed, they naturally lose weight, while others recognise that they binge and gain weight during difficult periods of their lives. Having awareness about this builds a person’s ability to protect against the impact of possible setbacks resulting from life stress on the resilience process. Another potential knock to resilience to be aware of for people with EDs is judgemental environments in terms of pressure, high expectations and comparisons (Sub-theme 6b). Many adults with EDs acknowledge that they may be overly-sensitive to these judgemental environments, but that there are also external factors that create these environments such as a family ethos of hard work, sibling rivalry, socio-economic status expectations, cultural expectations, social comparisons and stigma. Adults with EDs also appear susceptible to any factors that could potentially lead to a disturbed body image, which occurs when they are exposed to an over-emphasis on the importance of food and body image in society (Sub-theme 6c). Such factors include family members having a poor relationship with food or exercise, healthy eating obsessions among family or friends, comments made about the person’s weight or appearance and social media influencers who emphasise food or weight. These sub-themes of general life stress, judgemental environments and an emphasis on food and body image are areas which need to be monitored in order to prevent a set-back in the resilience process.

#### 3.4 Stage 3 of the resilience process: ‘I no longer need my ED’

During the final stage of the resilience process, adults with EDs no longer feel they need their ED in order to cope with the demands of the world or to survive due to better developed resilience and the utilisation of a wider set of resources (Stage 3: ‘I no longer need my ED’).

It is recognised that adults with EDs begin to identify resilience in themselves (Theme 7) and become aware that they no longer need the ED to cope and survive. The first piece of evidence that a person has entered ‘the resilient me’ stage is that they are able to reintegrate “more normal experiences” *(Client 6, female, aged 21-30)* into their lives (Sub-theme 7a)*.* Having structure and routine as well as social outlets assists in creating this sense of normality. During this stage, the adult with an ED desires to be free of ED symptoms including urges to participate in eating behaviours, “I’d like to eat normal and go out like normal people and not have to binge after” *(Client 10, female, aged 21-30).* Clinicians also speak about how routine and structure facilitates the implementation of treatment goals, as goals can be scheduled based on the person’s individual timetable. This stage is also defined by the adult with ED having developed a more positive mindset and future outlook on their life (Sub-theme 7b). This sub-theme includes personal factors that may be more traditionally perceived as ‘traits’ of resilience. Such factors include patience, determination, hope, high self-esteem, self-direction, and self-belief, all of which were noted by the participants as important factors leading to a more generally positive mindset. However, these factors are referred to as goals to work towards or characteristics to be built upon, and not factors that either exist or do not exist within a person. Once this positive mindset is achieved, a person is at a stage within the resilience process which better facilitates recovery (i.e. a reduction of ED symptoms, improved psychological well-being). However, clinicians warn that these personal factors can also work to maintain a person’s ED (i.e. self-determination to remain thin), and although adults with EDs might go on to recover successfully, some may otherwise be at risk of relapse. This highlights the dynamic process involved in developing resilience during ED recovery.
